# Acute Post-Bariatric Surgery Increase in Orexin Levels Associates with Preferential Lipid Profile Improvement

**DOI:** 10.1371/journal.pone.0084803

**Published:** 2014-01-06

**Authors:** Abhishek Gupta, Pierre Miegueu, Marc Lapointe, Paul Poirier, Julie Martin, Marjorie Bastien, Sunita Tiwari, Katherine Cianflone

**Affiliations:** 1 Centre de Recherche Institut Universitaire de Cardiologie & Pneumologie de Québec, Université Laval, Québec, Canada; 2 Department of Physiology, King George’s Medical University, Lucknow, India; 3 Faculté de pharmacie, Université Laval, Québec, Canada; CRCHUM-Montreal Diabetes Research Center, Canada

## Abstract

**Context:**

Orexin is a recently identified neuropeptide hormone.

**Objectives:**

Acute and long-term post-bariatric changes in Orexin and relationship to post-operative metabolic outcomes.

**Design and Participants:**

Men and women undergoing biliopancreatic diversion with duodenal switch bariatric surgery (n = 76, BMI≥35 kg/m^2^) were evaluated for body composition and plasma parameters at baseline, acutely (1 and 5 days) and long-term (6 and 12 months) post-surgery.

**Setting:**

University Hospital Centre, Canada.

**Interventions and Main Outcome Measures:**

Groups were subdivided based on acute (average 1 and 5 day) changes in Orexin prior to weight loss: (i)>10% Orexin decrease (n = 33, Orexin_DEC_) and (ii)>10% Orexin increase (n = 20, Orexin_INC_), to evaluate impact on long-term changes.

**Results:**

Both groups had comparable preoperative Orexin levels, BMI, age, sex distribution, diabetes and lipid lowering medication, plasma glucose and lipid parameters except for apolipoproteinB (p<0.007). Orexin increase was rapid and maintained throughout one year, while Orexin_DEC_ subjects remained significantly lower throughout. Over 12 months, changes in BMI, fat mass, and %fat mass were comparable. Fasting glucose and insulin increased immediately 1-day post-operatively, decreasing rapidly (5-day) and declining thereafter with the Orexin_INC_ group remaining lower than the Orexin_DEC_ group throughout (p = 0.001). Similarly, plasma cholesterol, triglyceride, LDL-C and HDL-C decreased at 1-day, increased slightly (5-day), except HDL-C, then decreased over 1 year, with greater decreases in Orexin_INC_ group relative to Orexin_DEC_ group.

**Conclusion:**

Rapid postoperative increases in plasma Orexin are associated with better improvement of glucose and lipid profiles following bariatric surgery.

## Introduction

With the present state of knowledge, it is difficult to treat and manage obesity using drug regimens. Currently, the best treatment method to achieve and sustain weight loss by inducing a negative balance between energy intake (or absorption) and energy expenditure, is bariatric surgery [1]. The weight loss obtained by bariatric surgery is primarily due to either a mechanical restriction of food intake (gastric banding) or a combination of restrictive and malabsorptive procedures (gastric bypass/biliopancreatic diversion). Bariatric surgery has become a common strategy used in the treatment of severely obese patients with a body mass index (BMI) >40 or >35 kg/m^2^ with co-morbidities. The effectiveness in improving abnormalities in insulin and glucose metabolism ranges from 48% with gastric banding to 99% with biliopancreatic diversion with or without duodenal switch [2], [3]. Interestingly, in restriction/malabsorption procedures, improved insulin sensitivity (“curing” diabetes) occurs before there is any significant weight loss, with studies reporting 80–100% remission rates within days (1 week) of surgery (Nandagopal 12:p671, 2010; Thomas 25:p175, 2010). Little is known of the exact mechanisms involved, but several have been proposed such as the “foregut hypothesis” and the “hindgut hypothesis” (Lifante 145:p549, 2008) which include physical anatomical changes, circulating hormonal changes and postoperative caloric restriction. As well, the potential involvement of neurohormonal feedback loops has been suggested [1]. Many peptide hormones have been recently recognized and a number of them, such as apelin, orexin, ghrelin, and leptin to name but a few, have been implicated in obesity pathophysiology, associated metabolic alterations and energy balance [4].

Orexin-A and B were identified in 1998 as endogenous ligands for the Orexin-1 (HCRT1) and Orexin-2 (HCRT2) G-protein coupled receptors [5] and both peptides originate from a single precursor produced by the prepro-orexin (PPO) gene. These peptides are identical to two hypothalamic peptides designated hypocretin-1 and hypocretin-2, that share a high degree of homology with the gastrointestinal peptide secretin [6]. Orexin-A is highly conserved among human, pig, dog, rat and mouse sequences whereas orexin-B differs in amino acid residues in rats and mouse from human. The structure of Orexin-A is more complex than that of Orexin-B, and Orexin-A may therefore be resistant to inactivating peptidases [7]. During the last decade Orexin receptors were identified within the lateral and posterior hypothalamic area as well as the enteric nervous system, adipose tissue, pancreas and the gut [8], [9], [10].

Orexin not only acts as an appetite stimulator, but also acts to regulate feeding behavior [11] which is linked to food intake, energy homeostasis and sleep [12]. Previous studies have shown that an increase in Orexin levels was related to an increase in food consumption and metabolic rate [13] [5], [15]. Orexin levels have also been shown to increase during low energy conditions and decrease when energy levels are high [5], [14]. In other words, soaring Orexin levels trigger wakefulness, vigilance and hunger; reduced levels induce inactivity and somnolence. In addition to promoting wakefulness and regulating food intake, Orexin-A has been implicated in diabetes mellitus and obesity [16], [17], [18].

Given the controversy surrounding the mechanisms involved in metabolic improvement prior to weight loss, identification of factors which change acutely (1–5 days) and are associated with subsequent improvements in metabolic profile (weight loss, glucose homeostasis or lipid profile) would provide valuable information on the functionality of these peptides as well as being useful as biomarkers. In the present study, we conducted a comprehensive investigation of short-term and long-term (up to one year) changes in plasma Orexin levels and the association with metabolic changes following biliopancreatic diversion with duodenal switch (BPD-DS) bariatric surgery.

## Materials and Methods

### Study Subjects

Subjects scheduled to undergo bariatric surgery (BPD-DS) were recruited through the bariatric surgery clinic of the Institut Universitaire de Cardiologie et de Pneumologie de Québec (IUCPQ), Laval University, Québec, Canada. Subjects were randomly selected (in chronological order of surgeries, regardless of diabetic status or current medication) for participation based on inclusion criteria: Subjects (male and female) >18 years of age, BMI ≥40 or BMI ≥35 kg/m^2^ with associated comorbidities, and surgeries were performed between 2006–2009. Subjects who had previously undergone bariatric surgery or those bearing a pacemaker were excluded (patients with a pacemaker cannot undergo electrical bioimpedance assessment). Only subjects who completed the study (5 time points, with blood samples collected) were included for subsequent biochemical analysis. Laboratory procedures were completed before statistical analysis was performed. The experimental protocol was approved by the ethics committee of the IUCPQ and all patients gave their written informed consent.

### Anthropometric Measurements

Subjects were assessed preoperatively and postoperatively (24 hours, 5 days, 6 months and 12 months). Blood samples were collected between July 2009 and May 2012. Height was measured using a stadiometer (SECA, 216 1814009, Brooklyn, NY, USA). Total body mass, body mass index (BMI), lean and fat masses were evaluated by electrical bio-impedance balance (Tanita TBF-310, Tokyo, Japan) following a 12-hour fast. BMI was calculated as weight (kg)/height (m^2^). Medical history was collected for diabetes, hypertension, coronary artery disease and dyslipidemia as well as the corresponding pharmacological therapy. The information provided by the patient was confirmed by consulting clinical files.

### Blood Collection and Biochemical Analysis

Venous blood was collected into EDTA containing tubes. Glycated hemoglobin (HbA1c) and fructosamine were evaluated in a fresh sample by turbidimetric inhibition immunoassay. All other tubes were rapidly placed on ice, centrifuged within minutes, plasma collected and frozen in aliquots at −80°C until analysis. Assays were measured in the hospital clinical biochemistry laboratory using standard methodology (fasting plasma glucose, cholesterol, triglyceride and HDL-cholesterol) [19] or in the research laboratory (Orexin, hsCRP and ApoB). LDL-cholesterol concentration was calculated using the Friedewald formula [20]. High sensitive C-reactive protein (hsC-RP) and Apolipoprotein B (ApoB) levels were measured by immunoturbidimetric method (Roche Diagnostics Integra 800 system). Plasma cholesterol, triglyceride (TG), HDL-Cholesterol (HDL-C) and glucose were measured using colorimetric enzymatic kits (Cholesterol, TG and HDL-C: Roche Diagnostics Indianapolis, IN, USA; glucose: Wako Chemicals, Richmond, VA, USA). Plasma insulin levels were measured by ELISA kit (Crystal Chem Inc, Downers Grove, IL, USA). Homeostatic model assessment of insulin resistance (HOMA-IR) was calculated from fasting plasma insulin and glucose levels as (insulin×glucose)/22.5, where the insulin concentration is reported as milli-units per liter and glucose as milli-molar concentrations.

### Plasma Orexin-A Levels

Plasma Orexin levels were measured by enzymatic immunoassay as per manufacturer’s instructions (RayBiotech, Cat# EIA-ORA-1, Lot# 0620196, Norcross, GA 30092). The minimum detectable concentration of Orexin-A is 98 pg/ml. The intra-assay coefficient of variation is 10% and inter-assay 15%.

### Statistical Analysis

All results are expressed as mean±s.e.m. as indicated. Preoperative values were compared between groups using unpaired Student t-test (normally distributed) or, for non-normally distributed variables, by Mann-Whitney test. Comparisons across different time periods were analyzed by repeated measures two-way ANOVA followed by Holm-Sidak post-hoc test. Prism (GraphPad Software Inc, La Jolla, CA, USA) and SigmaStat (Systat Software Inc, San Jose, CA, USA) software programs were used for graph and statistical analyses. Statistical significance was set at p value <0.05, where pNS indicates not significant.

## Results

### Baseline Characteristics of Subjects between Acute-Orexin-changes Groups

**Figure 1:**
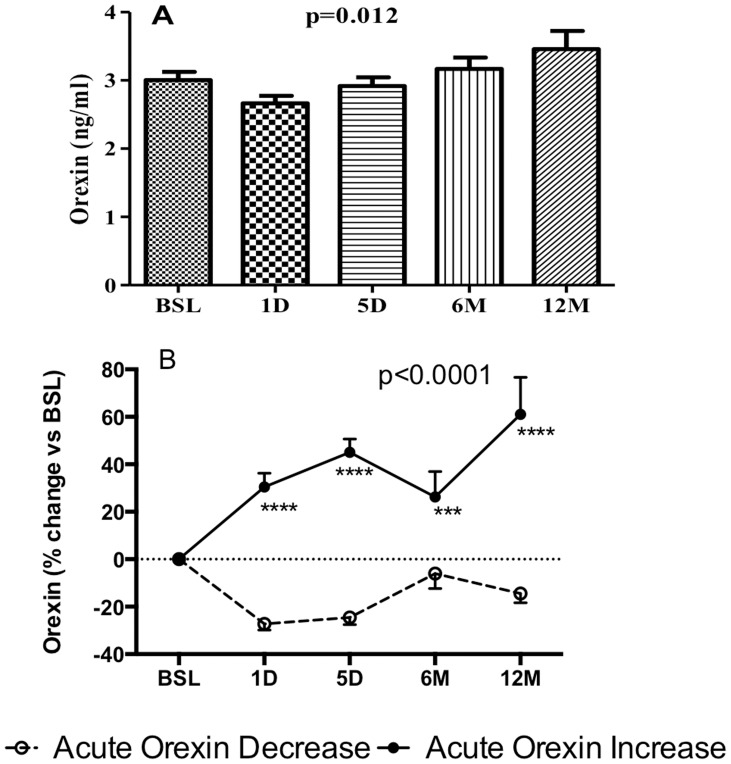
Plasma orexin levels (ng/ml) pre-operative (baseline; BSL) and post-operative at 1 and 5 days (D) 6 and 12 months (M) following BPD-DS bariatric surgery. Changes in plasma orexin levels at different time points (A), and % change of orexin between Orexin-DEC and Orexin-INC groups (B). Significance was determined by two-way repeated measures ANOVA followed by Holm-Sidak post-hoc test. Values are presented as mean ± s.e.m., where ***P<0.001 and ****P<0.0001 in Orexin-DEC vs. Orexin-INC groups.

Results for orexin levels for all 76 subjects over the one year time period are shown in Figure 1A, and indicate a significant change overall. However, when subjects were evaluated individually, there was a range in orexin response: orexin increased in some subjects, in other subjects orexin remained unchanged (±10%, within the coefficient of variation for the assay, n = 23) and in other subjects, orexin decreased. The orexin changes occurred as early as one day post-op, and remained consistent within each subject, such that the % change at 1 day correlated closely with % change at 5 days (r = 0.526, p<0.0001), 6 months (r = 0.410 p = 0.0006) and 12 months (r = 0.410 p = 0.0006). In order to focus on the impact of early (<1 week) changes in orexin, prior to any weight loss, the average of the 1 day and 5 day change in orexin was calculated. Using a 10% cut-point (which represents technical variability), groups were subdivided into >10% increase and >10% decrease. Baseline plasma Orexin levels were not significantly different between the two groups (3.31±0.31 vs. 2.92±0.28 ng/mL). As shown in Figure 1B, Acute-Orexin-Increase (Orexin-INC) subjects maintained that increase over the remaining time period (one year), while the Acute-Orexin-Decrease (Orexin-DEC) subjects maintained the decrease throughout.

The baseline pre-operative characteristics of the Orexin-INC (n = 20) and the Orexin-DEC (n = 33) groups are shown in Table 1. Both groups were of similar age, with comparable distribution of men and women, diabetics and non-diabetics. Further, both groups had comparable anthropometric variables as well as comparable fasting plasma parameters related to glucose homeostasis and lipid profile. Only pre-operative apolipoprotein B levels were different between the two groups (p = 0.007).

**Table 1:** Baseline parameters in groups separated based on average of 1-Day and 5-Day acute Orexin changes.

Parameters	ACUTE-OREXIN DECREASE (n = 33)	ACUTE-OREXIN INCREASE (n = 20)	P value
Orexin (ng/mL)	3.31±0.31	2.92±0.28	pNS
*Distribution of subjects*			
Male/Female	10/22	8/12	NS
Diabetic/Non-diabetic	15/18 (45%)	11/9 (55%)	NS
Lipid Lowering Therapy	15/18 (45%)	10/10 (50%)	NS
*Anthropometric variables*			
Age (years)	44.2±2.1	38.3±2.1	NS
Weight (kg)	136.6±3.8	130.6±6.9	NS
BMI (kg/m^2^)	50.5±1.0	49.9±1.9	NS
Fat Mass (kg)	70.8±2.4	67.4±4.4	NS
Lean Mass (kg)	65.8±1.8	63.2±3.1	NS
Fat Percentage (%)	51.7±0.7	51.5±1.2	NS
*Glucose homeostasis*			
Glucose (mmol/L)	6.31±0.41	7.31±0.68	NS
Insulin (pmol/L)	214±19	159±18	NS
HOMA-IR	9.17±1.3	7.83±1.2	NS
Fructosamine (µmol/L)	214±6.6	228±10.7	NS
HbA1c (%)	0.060±0.002	0.064±0.003	NS
*Lipid profile*			
Cholesterol (mmol/L)	4.6±0.15	5.1±0.21	NS
Triglyceride (mmol/L)	1.53±0.12	1.95±0.34	NS
LDL-C (mmol/L)	2.59±0.13	2.91±0.14	NS
HDL-C (mmol/L)	1.29±0.04	1.31±0.09	NS
*Biochemical variables*			
**ApoB (g/L)**	**0.74**±0.03	**0.87**±0.03	**0.007**
hsCRP (mmol/L)	7.36±0.95	16.1±6.5	NS

The values are presented as mean±s.e.m., where significant differences were analyzed by Student’s t-test (or Mann-Whitney for non-normally distributed variables) for Acute-Orexin-Decrease vs Acute-Orexin-Increase, where “p value” indicates significance and p>0.05 is non-significant (NS). For distribution of subjects between male/female, diabetic/non-diabetic and lipid lowering therapy, results were analyzed by χ^2^ test. Abbreviations: ApoB: Apolipoprotein B; BMI: body mass index; HbA1c: Hemoglobin A1c; HOMA-IR: Homeostatic model assessment index-insulin resistance; hsCRP: high-sensitivity C-reactive protein; LDL-C and HDL-C: low density and high density lipoprotein cholesterol.

### Changes in BMI and Body Composition Following BPD-DS Surgery

**Figure 2:**
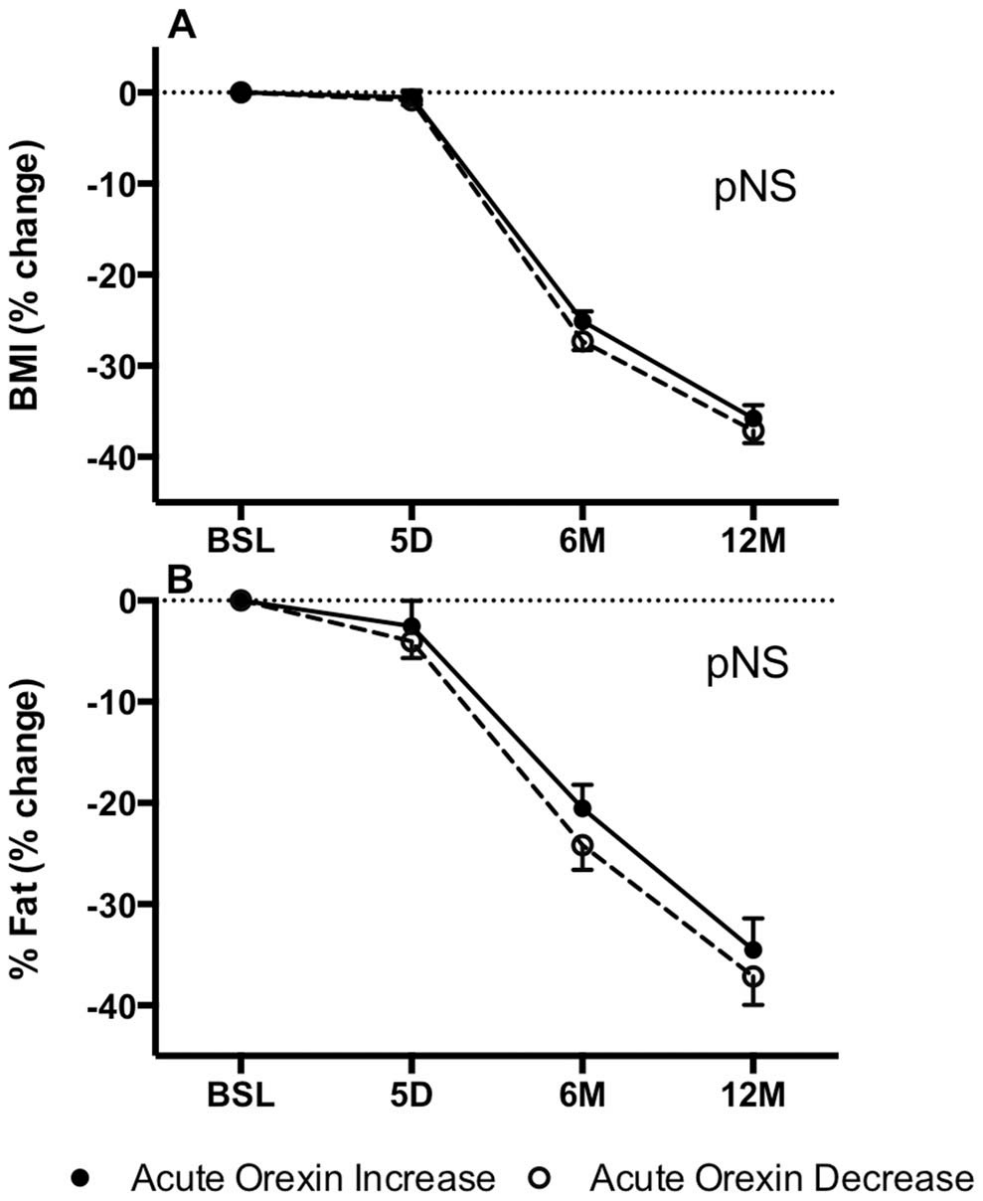
Changes in BMI and body composition after BPD-DS bariatric surgery. Changes in body mass index (BMI) (% change, A), and % fat (% change, B) at 5 days, 6 and 12 months between Orexin-INC and Orexin-DEC groups. There were no significant differences between the two groups by two-way repeated measures ANOVA followed by Holm-Sidak post-hoc test. Values are presented as mean ± s.e.m.

Within the first few days following the surgery (up to 5 days), there was no significant change in body composition between Orexin-INC and Orexin-DEC groups (Figure 2A and 2B). However, at 6 months, there were marked decreases in BMI in both groups by 25–27%, reflecting a decrease in both fat mass (22–24%, Fig. 2B) and lean mass (data not shown), but with comparable changes in both groups. This was also true at 12 months, with further decreases in BMI (36% to 37%) and percent body fat (average 36%) although the patient weight status still remained within the obese range.

### Rapid Change in Glucose Homeostasis

**Figure 3:**
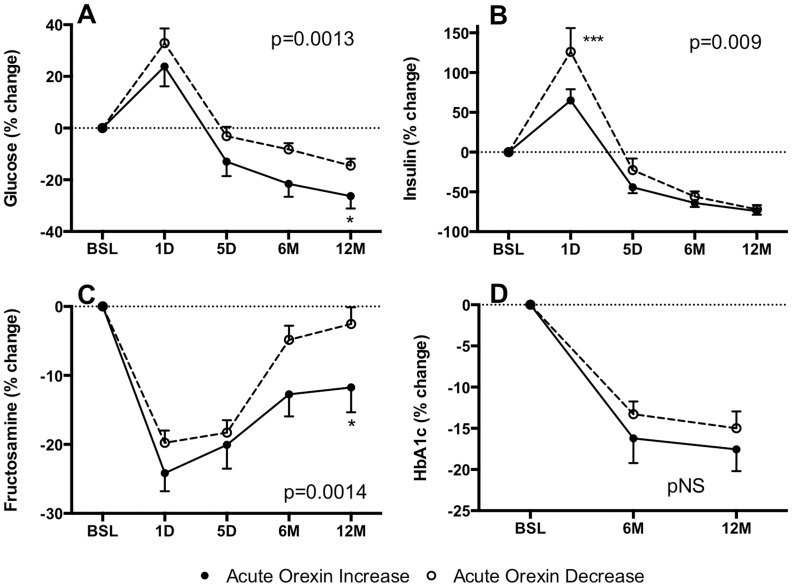
Rapid changes in glucose homeostasis after BPD-DS bariatric surgery. Results are presented for glucose (A), insulin (B), fructosamine (C) and HbA1c (D) in Orexin-INC and Orexin-DEC groups after surgery. Significance was determined by two way repeated measures ANOVA followed by Holm-Sidak post-hoc test. Values are presented as mean ± s.e.m. where *P<0.05 and ***P<0.001.

Out of 33 Orexin-DEC subjects, 15 (45%) were diabetic as indicated in Table 1. In the Orexin-INC group, of 20 patients total, 11 (55%) were diabetic, similar to the other group. Post-operatively, over time, there was a marked reduction in the diabetic status, but there was no difference between groups. However, although changes in weight and % fat mass were comparable between both groups, the response to various glucose and lipid parameters was not the same. Fasting glucose increased significantly immediately post-operatively (1D), decreasing rapidly (5D) thereafter, then moderately for up to one year (12M) (Figure 3A). However in the Orexin-INC group, the rapid glucose increase was not as pronounced, and the subsequent values remained significantly lower as compared to the Orexin-DEC group (p = 0.0013). This improved glucose profile in the Orexin-INC group was achieved in the presence of relatively lower insulin levels (which increased, then decreased post-operatively, Figure 3B, p = 0.009 between groups). Fasting fructosamine levels decreased acutely at day 1 then partially rebounded, although remaining lower than baseline at 5 days, 6 months and 12 months post-bariatric surgery (Figure 3C), again with lower relative levels in the Orexin-INC group (p = 0.0014). While HbA1c decreased over time, although there was no significant difference between the two groups (Figure 3D).

### Rapid Improvement in Lipid Profile

**Figure 4:**
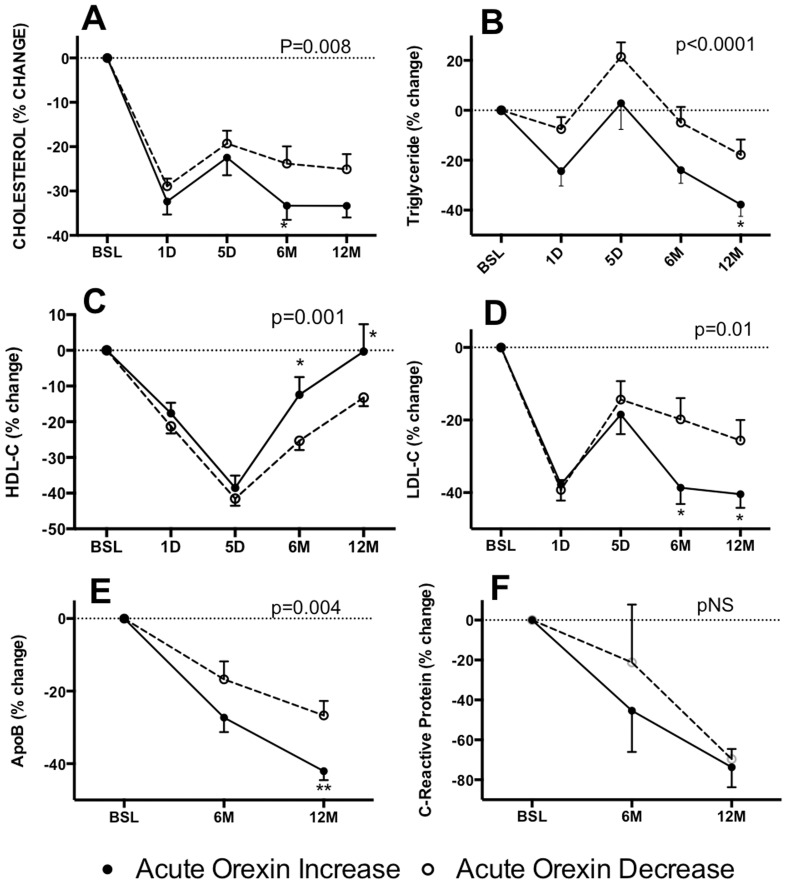
Rapid and long-term improvement in lipid profile after BPD-DS bariatric surgery. Changes in plasma total cholesterol (A), triglyceride (B), HDL-C (C), LDL-C (D), ApolipoproteinB (E), and C-Reactive Protein (F) were found at 6 and 12 months between Orexin-INC and Orexin-DEC groups. Significance was determined by two-way repeated measures ANOVA followed by Holm-Sidak post-hoc test. Values are presented as mean ± s.e.m., where *P<0.05, and **P<0.01.

In the Orexin-DEC group at baseline, 15 (45%) were being treated with lipid lowering therapy, while 10 (50%) patients were being treated in the Orexin-INC group (Table 1). Postoperatively, there was a reduction in those being treated, which was comparable between the two groups. However, there were both acute (1 and 5 days) and long-term (6 and 12 months) changes in lipid profiles, with overall greater changes in the Orexin-INC group relative to the Orexin-DEC group (Figure 4). Specifically, plasma cholesterol, triglyceride, LDL-C and HDL-C decreased at day 1 and increased at 5 days (except HDL-C) in both groups, at which point none were on lipid-lowering therapy. However the Orexin-INC group had significantly greater decreases in plasma total cholesterol (Figure 4A, p = 0.008), plasma triglyceride (Figure 4B, p<0.0001), and plasma LDL-C (Figure 4D, p = 0.01) and maintained higher plasma HDL-C (Figure 4C, p<0.001). Over the long-term (at 6 months and 12 months), there was a significant continued reduction of fasting cholesterol, triglyceride and LDL-cholesterol, again with greater changes in the Orexin-INC group, while HDL-C increased (Figure 4C).

### Long Term Changes in Apolipoprotein B and hsC-Reactive Protein Levels

Over the long term (at 6 months and 12 months), ApoB levels were significantly decreased at both 6 months and 12 months, with significantly greater decreases in the Orexin-INC group (Figure 4E, p = 0.004). By contrast, the inflammatory marker hsCRP was decreased comparably at 6 and 12 months in both groups (Figure 4F, p = NS).

## Discussion and Conclusion

In the present study, we performed a comprehensive investigation of metabolic changes and their acute and long-term (up to one year) effects in severely obese patients following BPD-DS surgery. This type of bariatric surgery is well known for its long-term effects on weight loss and improvements in metabolic profile, although not all patients improve to the same degree [21]. Interestingly, even before acute weight loss, during the early post-operative period (<1 week), subjects benefit from the surgical intervention with a reduction in overt diabetic status and improved insulin sensitivity within days of bariatric surgery and this, before any observed weight loss [22]. However, important questions still remain such as (i) the mechanism for the rapid resolution of diabetes in the early post-operative period, even before weight loss and (ii) an explanation for the variability in subsequent metabolic profile in spite of comparable weight loss. Thus, identifying early post-operative changes, and the relation to subsequent metabolic improvements could provide important biomarkers for targeting. The major novel findings of our present investigation are, first, that there are early acute changes in orexin levels prior to weight loss, and these changes are present in some but not all patients. Further, the early orexin changes were maintained consistently within subjects throughout the observation period (one year). Lastly, notwithstanding comparable long-term weight decreases, the early changes in orexin are associated with differential improvements in lipid and glucose profiles throughout the one-year postoperative period.

The role of orexin, a hypothalamic neuropeptide, in obesity and weight loss in general, and in post-bariatric surgery in particular, is still unclear. To the best of our knowledge, the current study is the first study evaluating Orexin-A levels in BPD-DS patients with both acute and long-term follow-up. Previous studies have reported that baseline orexin levels were lower in obese individuals compared with healthy controls [23], [24], [25] and can increase overall during weight loss [26]. However in the present study, in the acute post-operative phase (1 day) and prior to any weight loss, some subjects demonstrated an increase in orexin while others decreased their plasma orexin levels. This raises the question as to what factors regulate these acute changes in orexin during the acute post-operative phase.

As reviewed recently [27], [28], chronic states of obesity, ageing and depression, as well as narcolepsy, traumatic brain injury and lipopolysaccharides (LPS) are all associated with reduced orexin levels. Increased orexin levels are associated with pregnancy, chronic obstructive pulmonary disease, obstructive sleep apnea-hypopnea syndrome and hemodialysis [27], [29], [30]. However, none of these features are likely responsible for the acute changes in orexin in the present study. On the other hand, food restriction, amino acids, gut hormones (such as ghrelin and glucagon-like peptide-1 (GLP-1)), and high-fat diets, are all known to increase orexin, and any of these components could potentially play a role in the acute post-operative changes in orexin seen here [31], [27]. Some studies have suggested that the rapid resolution of insulin resistance and hyperglycemia that appear before observable weight loss may be related to alterations in gut regulatory peptides produced by the surgical procedure [32], which might, in turn, also influence orexin levels. Ghrelin, a gut peptide, increased the reward value of a high-fat diet, and this in an orexin-dependent manner [33].

Glucose levels may play a particularly interesting role in regulating orexin levels in the present study. Hypoglycaemia increases orexin in neurons, and higher glucose levels in the cerebrospinal fluid can suppress orexin; by contrast, circulating glucose increases orexin release from the pancreas [31], [27]. These differential effects of glucose may relate to the pattern of Orexin-INC or Orexin-DEC noted in our subjects, a pattern that is also reflected by differential glucose, insulin and fructosamine levels between these two groups, and a difference that was maintained throughout the one-year follow-up period. With respect to glucose, the higher orexin coupled to lower glucose and insulin may relate to an interesting feedback regulation. While glucose increases both insulin and orexin secretion from the pancreas [27], orexin suppresses insulin secretion [34], and this may be an effect that is maintained throughout the one-year follow-up period.

In terms of function, orexin has been shown to increase glucose uptake and promote insulin-induced glucose uptake and glycogen synthesis in skeletal muscle by activating the sympathetic nervous system [35], as well as protected against development of peripheral insulin resistance induced by ageing or high-fat feeding in mice [36].

Finally, in adipose tissue, orexin has direct *in vitro* effects on glucose uptake, lipid accumulation and adiponectin secretion in mouse 3T3 and rat adipocytes [37] and the presence of functional orexin receptors has been demonstrated in human adipose tissue, suggesting a role for orexins in adipose tissue metabolism and adipogenesis [9]. Further, central administration of orexin has biphasic effects on adipose tissue lipolytic activity mediated through sympathetic nerve activity [38]. Conversely, in orexin-deficient mice, there are abnormalities in energy homeostasis, with insulin resistance in the hypothalamus and liver with ageing [39], [40]. Altogether, these data support the proposed hypothesis that in the long-term, altered orexin may promote obesity resistance [31].

The strengths and limitations of this study should be noted. In the present study, there was no comparison to a weight loss group induced by non-surgical means, although diet studies rarely achieve weight and fat mass loss of this magnitude. Further, all of the subjects in the current study were severely obese, and even at one-year following surgery, the body size of these subjects would still fall within the obese range. Therefore there were no non-obese surgical patients evaluated over a similar time range. Finally, the associated changes can only be correlative, and are not proof of cause and effect. On the other hand, these subjects were thoroughly evaluated at multiple time points (five time points), with a comprehensive metabolic and physical evaluation, allowing intra-individual comparison of changes over 1 year.

In conclusion, the results of this study indicate that an acute post-bariatric surgery increase in orexin levels is associated with a maintenance of increased orexin throughout the one-year follow-up period, and predicts a better lipid profile improvement. This post-operative increase in orexin levels is also associated with rapid improvement in glucose metabolism. The present study provides valuable information on potential biomarkers for targeting in therapy.
